# Screening children and adolescents for cutaneous malignant melanoma: the impossible trade-off between life-years saved and unnecessary biopsies

**DOI:** 10.3389/fonc.2026.1726375

**Published:** 2026-02-23

**Authors:** Lauro Bucchi, Silvia Mancini, Pietro Ceretti, Federica Zamagni, Emanuele Crocetti, Luigino Dal Maso, Stefano Ferretti, Flavia Baldacchini, Orietta Giuliani, Alessandra Ravaioli, Rosa Vattiato, Giuliano Carrozzi, Maria Michiara, Antonino Musolino, Fabio Falcini, Ignazio Stanganelli

**Affiliations:** 1 Emilia-Romagna Cancer Registry, Romagna Cancer Institute, Istituto di Ricovero e Cura a Carattere Scientifico (IRCCS) Istituto Romagnolo per lo Studio dei Tumori (IRST) Dino Amadori, Meldola, Italy; 2 University of Parma, Parma, Italy; 3 Cancer Epidemiology Unit, Centro di Riferimento Oncologico di Aviano (CRO) Istituto di Ricovero e Cura a Carattere Scientifico (IRCCS), Aviano, Italy; 4 Dipartimento di Medicina Translazionale e per la Romagna, University of Ferrara, Ferrara, Italy; 5 Emilia-Romagna Cancer Registry, Modena Unit, Public Health Department, Local Health Authority, Modena, Italy; 6 Emilia-Romagna Cancer Registry, Parma Unit, Medical Oncology Unit, University Hospital of Parma, Parma, Italy; 7 Breast & GYN Unit, Medical Oncology, Istituto di Ricovero e Cura a Carattere Scientifico (IRCCS) Istituto Romagnolo per lo Studio dei Tumori (IRST) Dino Amadori, Meldola, Italy; 8 Department of Medical and Surgical Sciences, University of Bologna, Bologna, Italy; 9 Cancer Prevention Unit, Local Health Authority, Forlì, Italy; 10 Skin Cancer Unit, Istituto di Ricovero e Cura a Carattere Scientifico (IRCCS) Istituto Romagnolo per lo Studio dei Tumori (IRST) Dino Amadori, Meldola, Italy; 11 Dermatology Clinic, Department of Medicine and Surgery, University of Parma, Parma, Italy

**Keywords:** adolescents, biopsy, children, cutaneous malignant melanoma, mass screening, overtreatment

## Abstract

**Introduction:**

In Europe, insufficient data exist to assess the magnitude and results of screening practice for cutaneous malignant melanoma (CMM) among children and adolescents. In this population-based study covering part of the Emilia-Romagna Region (northern Italy), multiple indicators of screening for CMM by patient age were evaluated.

**Methods:**

The current population of the study area is over 2,600,000. The records of patients with CMM (2003-2017) were extracted from the Emilia-Romagna Cancer Registry. The records of dermatologic office visits and skin biopsies were downloaded from the outpatient healthcare database of the Regional Administration. Patient age was grouped as 0-19 (children and adolescents), 20-39, 40-59, 60-79, and ≥80 years. The study endpoints were in situ/invasive CMM incidence rate, Breslow tumor thickness distribution, mortality rate, dermatologic office visit rate, skin biopsy rate, number (of patients) needed to visit (NNV) and biopsy (NNB) to detect a case of disease, and the potential number of life-years saved, equivalent to the number of years of life expectancy left at diagnosis.

**Results:**

Data for 11,679 patients, 4,593,988 dermatologic office visits and 849,343 skin biopsies were obtained. Patients aged 0-19 years (n=51) accounted for 0.4% of total incident CMM cases, 0.3% of total deaths from CMM, and 1.4% of total potential number of life-years saved. The annual dermatologic office visit rate at age 0-19 years was 9.2%. The NNV was 11,362.2 at age 0-19 years versus 305.6 in the middle-aged group of 40-59 years (ratio, 37.2). The NNB was 681.5 and 66.4, respectively (ratio, 10.3). The total potential number of life-years saved was 2939.9 versus 98,382.2, respectively (ratio, 0.03).

**Conclusion:**

When screening children and adolescents for CMM, a trade-off between life-years saved and unnecessary biopsies is impossible to make because of the minimal prevalence and the ill-defined clinical/dermoscopic features of the disease.

## Introduction

In the greater part of developed countries, screening for cutaneous malignant melanoma (CMM) with visual skin examination is not recommended by health agencies and expert panels (1) and is not implemented as a standard of care. Consequently, it is delivered on a spontaneous basis, where patients initiate the process, or an opportunistic basis, where screening is offered to individuals who present to healthcare providers for other reasons (2, 3).

In the past decades, the increase in the incidence of CMM occurring in virtually all Caucasian populations of the world (4, 5) has been attributed, in varying proportions, to a phenomenon of overdiagnosis. This would be secondary to an increased diagnostic scrutiny, that is, a combination of more screening skin examinations, lower threshold to perform skin biopsy, and lower pathologic threshold to interpret the histologic changes observed as malignant (6).

More recent –and still insufficient– data have suggested that this unplanned practice has evolved to include children and adolescents with increasing biopsy and excision rates (7–13). The value of preventing a death depends on the estimated number of life-years gained. By implication, early detection of CMM in young persons is a naturally attractive goal. In addition, the past increasing incidence trend in young adults has boosted the attention of dermatologists towards this subset of the population. A third factor has been the increasing level of general alarm for the social impact of the disease. Noteworthy, the public concern has been indirectly intensified by effective education campaigns for the avoidance of ultraviolet radiation exposure (14).

In the first two decades of life, however, the absolute risk of CMM is extremely low (15). This, coupled with the elevated life expectancy of young people, greatly decreases the risk of overdiagnosis. Conversely, a high degree of diagnostic scrutiny placed on skin changes in children and adolescents leads to unnecessary healthcare costs, unnecessary biopsies, overtreatment, and patient morbidity.

Because of these concerns, the peculiarities of clinical presentation of pigmented skin lesions in the early ages have attracted more research in order to improve the accuracy of diagnostic decisions (16–19). From the epidemiologic perspective, instead, the data for quantifying the size and consequences of screening for CMM in young people are still scarce worldwide, but particularly in Europe.

This study is part of a broader research project aimed at investigating the recent epidemiologic trends of CMM in Italy (14, 20–22). Here, we have addressed the screening experience of the resident population of the Emilia-Romagna Region (northern Italy) by patient age. Our endpoints included *in situ* and invasive CMM incidence rate, Breslow tumor thickness distribution, mortality rate, dermatologic office visit rate, skin biopsy rate, number (of patients) needed to visit (NNV) and biopsy (NNB) to detect a case of disease (12, 23), and potential number of life-years saved, equivalent to the number of years of life expectancy left at diagnosis.

## Materials and methods

### Population and incidence of CMM

From 2003 to 2017, the total population of the Emilia-Romagna Region has grown from 2,394,732 to 2,626,263. The number and percentage of people aged 0-19 years have increased from 375,918 or 15.7% to 460,573 or 17.5%.

According to the European Cancer Information System, a web application that integrates data from population-based cancer registries (24), the average annual age-standardized (2013 European Standard Population) incidence rate of CMM between 2010 and 2019 was 25.4 per 100,000. Children and adolescents, that is, people aged 0-19 years according to the World Health Organization criteria (25), had an age-standardized (by 5-year age groups) incidence rate of 0.5 per 100,000. Both figures were roughly intermediate in the range of European national or regional rates (9.2 to 44.3 and 0.1 to 0.8 per 100,000, respectively).

### Sources of data

The acquisition of data for this study has been validated in two previous studies in which the same sources of information were used (21, 22). Incidence records (2003-2017) were extracted from the database of the Emilia-Romagna Cancer Registry. The Registry was established with the merging of six pre-existing local registries. In four of the six registration areas (the provinces of Parma, Modena and Ferrara and the sub-region of Romagna), the data collected fulfilled the following eligibility criteria: (1) they covered ≥ 10 consecutive years; (2) they included incidence both of situ and invasive CMM; and (3) they included Breslow tumor thickness information for at least 75% of invasive CMM cases on an annual basis.

The data extraction was done using the International Statistical Classification of Diseases and Related Health Problems, 10th revision (ICD-10), codes D03.0 to D03.9 (*in situ* CMM) and C43.0 to C43.9 (invasive CMM) (26). Deaths attributable to CMM were classified using both the International Classification of Diseases, 9th revision (ICD-9), codes 172.0 to 172.9 (27) and the ICD-10 codes C43.0 to C43.9.

Then, we accessed the outpatient healthcare database of the Emilia-Romagna Regional Administration (ASA database), where the individual electronic records of services delivered in outpatient clinics of public health facilities are stored for administrative purposes. The records of dermatologic office visits and skin biopsies were downloaded using 3-digit and 4-digit codes from the International Classification of Diseases, 9th Revision, Clinical Modification (28), other modified 5-digit codes created by the Department of Health, and combinations of codes. Multiple skin biopsies from a single patient were all considered eligible for analysis.

More details on the criteria used to select and download the ASA records can be found in one of the abovementioned previous articles (22).

### Statistical methods

According to the WHO criterion, adolescents were defined as people in the age group of 10–19 years (25).

Invasive CMM cases were categorized by Breslow tumor thickness using a simplified criterion from the 8th edition of the American Joint Committee on Cancer (AJCC) staging system (≤1.0, >1.0) (29).

The average annual office visit rate and the average annual skin biopsy rate were obtained by summing up the annual number of visits and biopsies and the annual populations for the entire study period. All rates were age-standardized to the 2013 European standard population. The NNV and the NNB were calculated by dividing the total number of dermatologic visits and, respectively, the total number of biopsies over the study period by the number of CMMs detected. The 95% confidence intervals around the NNV and the NNB were calculated with the ‘delta’ method (30, 31).

The potential number of life-years saved by detection and treatment, or the number of life-years saved under the assumption that the disease, if left untreated, would be fatal, was calculated for each patient by subtracting its age at diagnosis from the sex-specific life expectancy at birth of the population of the Emilia-Romagna Region. Life expectancy data were downloaded from the website of the Italian National Institute of Statistics (ISTAT) (32). The ISTAT calculates life expectancy at birth as the average number of years a person can expect to live from birth, assuming that he/she is exposed throughout lifetime to the age-specific mortality risks observed in the reference year (33). We used the life expectancy data estimated for the year 2024. When the difference between life expectancy at birth and age at diagnosis was negative, the potential number of life-years saved was set to zero. The total potential number of life-years saved was obtained by summing up the individual numbers of life-years saved of all patient in each age group.

For comparison purposes, we used the middle-aged group of 40-59 years as a reference category for the total potential number of life-years saved, the NNV and the NNB at age 0-19 years. Data analysis was done with the Stata statistical package, Release 15.1 (StataCorp, College Station, TX, USA).

## Results

### Patient characteristics

We studied a total of 11,679 patients, including 3453 patients with *in situ* CMM and 8226 (70.4%) patients with invasive disease. Their median age at diagnosis was 61 and 60 years, respectively. Information on Breslow tumor thickness was available for 7736 (94.0%) patients with invasive CMM, with a median measurement of 0.70 mm. The case series was composed of 5982 males (51.2%) and 5697 females.

From the ASA database, we extracted the records of 4,593,988 dermatologic office visits and 849,343 skin biopsies. The median patient age was 51 and 55 years, respectively. During the study period, 1251 deaths from CMM were registered (median patient age, 72 years).

### Incidence and mortality by patient age

**Table 1** shows the incidence and mortality data and the median Breslow tumor thickness of incident CMM cases by patient age. Among patients aged 0-9 year, a single case of disease was detected. The 51 total cases detected between 0 and 19 years of age accounted for 0.4% of the 11,679 total incident cases. The age distribution of *in situ* and invasive CMM is further illustrated by **Supplementary Figure S1**, where the curves of average annual incidence rates by sex and 5-year age group are shown.

**Table 1:** Number of cases and incidence rates of *in situ* and invasive cutaneous malignant melanoma, Breslow tumor thickness distribution, number of cause-specific deaths and cause-specific mortality rates.

Age in years	Number of cases			Average annual incidence rate*			Median Breslow tumor thickness in mm (range)	Number of deaths	Average annual mortality rate*
	In situ	Invasive	Total	In situ	Invasive	Total			
0-9	0	1	1	0.0	0.0	0.0	4.00†	1	0.0
10-19	8	42	50	0.3	1.4	1.6	0.79 (0.10-7.60)	3	0.1
Subtotal	8	43	51	0.1	0.7	0.8	0.79 (0.10-7.60)	4	0.1
20-39	467	1223	1690	4.7	12.2	16.9	0.60 (0.00-30.00)	54	0.5
40-59	1135	2832	3967	10.3	25.5	35.8	0.62 (0.00-50.00)	237	2.2
60-79	1473	3025	4498	17.6	36.1	53.7	0.80 (0.00-85.00)	577	6.8
≥80	370	1103	1473	14.0	42.0	56.0	1.80 (0.07-48.00)	379	14.6
Total	3453	8226	11,679	8.3	19.8	28.1	0.70 (0.00-85.00)	1251	2.9

*Per 100,000, age-standardized to the 2013 European standard population; †Breslow tumor thickness of the only case detected in this age group. Males and females combined. Emilia-Romagna Region (northern Italy), 2003-2017.

In the age range 0-19 years, the median Breslow tumor thickness was 0.79 mm, virtually the same figure as observed among patients aged 60-79 years (0.80 mm). The proportion of invasive CMMs >1.0 mm thick was, respectively, 34.9% and 43.7%.

The 4 deaths observed between 0 and 19 years of age accounted for 0.3% of the total 1251 deaths from CMM.

### Dermatologic office visits and skin biopsies by patient age

**Table 2** shows the number and the average annual rates of dermatologic office visits and skin biopsies, the NNV and the NNB by patient age. The annual dermatologic office visit rate at age 0-19 years was 9.2% (overall, 11.9%). Patients aged 0-9 years had the lowest rate. Having a prevalence of one case only, however, they experienced a NNV as high as 222,849. For the whole age group of 0-19 years, the NNV was 11,362.2. This number was 37.2 times higher than in the middle-aged group of 40-59 years (305.6). The NNB was 681.5 and 66.4, respectively, for a ratio of 10.3.

**Table 2:** Number of dermatologic office visits and skin biopsies, average annual dermatologic office visit and skin biopsy rate, and number of patients needed to visit and to biopsy to detect a case of in situ/invasive cutaneous malignant melanoma, by patient age.

Age in years	Number of office visits	Average annual office visit rate*	NNV (95% CI)	Number of skin biopsies	Average annual skin biopsy rate*	NNB (95% CI)
0-9	222,849	6.7	222,849.0 (31,390.3-1,582,071.0)	4641	0.1	4641.0 (653.9-32,941.0)
10-19	356,620	11.5	7132.4 (5405.8-9410.4)	30,118	1.0	602.4 (456.7-794.6)
Subtotal	579,469	9.2	11,362.1 (8635.1-14,950.2)	34,759	0.6	681.5 (518.0-896.6)
20-39	1,021,897	11.0	604.7 (576.6-634.2)	181,088	1.9	107.2 (102.2-112.4)
40-59	1,212,264	10.9	305.6 (296.3-315.2)	263,262	2.4	66.4 (64.4-68.5)
60-79	1,363,400	16.3	303.1 (294.4-312.1)	278,676	3.3	62.0 (60.2-63.8)
≥80	416,958	15.9	283.1 (269.0-297.9)	91,558	3.5	62.2 (59.1-65.4)
Total	4,593,988	11.9	393.4 (386.3-400.6)	849,343	2.1	72.7 (71.4-74.0)

NNV, number of patients needed to visit to detect a case of in situ/invasive cutaneous malignant melanoma. NNB, number of patients needed to biopsy to detect a case of in situ/invasive cutaneous malignant melanoma. CI, confidence interval. *Percent, age-standardized to the 2013 European standard population. Males and females combined. Emilia-Romagna Region (northern Italy), 2003-2017.

### Life-years saved

**Table 3** shows the potential number of life-years saved by patient age. The median number decreased with increasing patient age. The total number was 2939.9 at age 0-19 years (1.4% of the potential number of life-years saved in the whole patient population) and peaked at 98,382.2 among those who had 40-59 years of age at diagnosis. The ratio between these two numbers was 0.03.

**Table 3:** Median and total potential number of life-years saved, by patient age.

Age in years	Number of life-years saved	
	Median (range)	Total
0-9	81.9 (81.9-81.9)	81.9
10-19	67.4 (63.3-75.9)	2858.0
Subtotal	67.9 (63.3-81.9)	2939.9
20-39	50.3 (43.3-65.9)	62,990.9
40-59	34.9 (23.3-45.9)	98,382.2
60-79	14.3 (3.3-25.9)	43,232.7
≥80	0.0 (0.0-5.9)	1459.7
Total	24.3 (0.0-81.9)	209,005.4

Males and females combined. Emilia-Romagna Region (northern Italy), 2003-2017.

## Discussion

### Interpretation

We assume that the average annual proportion of residents who underwent a dermatologic office visit, 11.9%, included both self-selected individuals (directly attending dermatologic offices) and individuals previously selected by primary care physicians. If so, this means that the total annual population screened for CMM was larger than it appears from our data.

With respect to children and adolescents, the process was inaccurate and inefficient to an extreme degree. There are two main reasons for this. The first is that the prevalence of CMM at these ages is minimal. On the one hand, this decreases substantially the risk of overdiagnosis, which is also moderated by the life expectancy of young people. On the other hand, however, unnecessary healthcare utilization and overtreatment increase accordingly, with a NNV and particularly a NNB unacceptably high. The observed NNB at age 0-19 years, 681.5, is 10-fold higher than at age 40-59 years. In addition, a minimal prevalence of disease has a detrimental effect on the benefit that primary care physicians and office-based dermatologists expect from their strategy, even though not supported by experimental evidence. Screening children and adolescents aims, supposedly, to maximize the number of life-years saved. In fact, this mission can be accomplished at the individual patient level, because the potential number of life-years saved is greater for a young patient, but not at the general population level. This is because the sum of the individual numbers of life-years saved is enormously greater among adult patients, with a peak between 40 and 59 years of age. In brief, when screening children and adolescents for CMM, a trade-off between life-years saved and unnecessary biopsies is impossible to make.

The second reason for the inefficiency of screening for CMM among children and adolescents is that the impact on Breslow tumor thickness of incident CMM cases, in our data, is far from satisfactory. The median measurement of cases diagnosed at age 0-19 years was virtually the same as observed among patients aged 60-79 years. The ill-defined clinical and dermoscopic features of many CMMs in younger patients do probably account for the diagnostic delays. Indeed, studies have been published suggesting that CMMs arising in the first two decades of life have distinct characteristics (34–36).

### Comparison with the literature

We are not aware of any previous study reporting comparable population-based data from people aged 0-19 years living in Europe. Three studies have considered single-center clinical case series. The number needed to excise (NNE) varied from 259 in an Italian study (13) to 593.8, with a peak of 1141 at age 10-14 years, in an Austrian study (11). In a case series of 996 patients from Spain, no CMM was detected (10).

In a large study from the U.S., the NNB was 982, with a peak of 1896 at age 0-9 years (12). In three Australian studies (7–9), the younger age groups had the highest NNE but the figures were substantially lower than those seen in Europe and U.S., with a range of 83 (8) to 145.9 (7). This variability, albeit partly explained by different risks of melanocytic lesions and different healthcare systems, underscores the obvious fact that the criteria used to decide for skin biopsy in children and adolescents are non-standard.

The American study of Oliveria et al. (12), given its large sample size and the use of our own endpoint, is particularly well-suited for comparison. Our NNB for total patients aged 0-19 years was appreciably lower (681.5 versus 982) whereas our peak at age 0-9 years was almost 2.5-fold higher (4641 versus 1896).

### Implications for health policy, research and information

Inappropriate diagnostic assessment of pigmented skin lesions can be countered with a combined strategy of audit, healthcare planning, research and information. Pediatric skin disorders undergoing biopsy and excision are seldom audited in the clinical practice. Instead, they should be routinely reviewed focusing on the indication (17). A multidisciplinary approach can be expected to improve the appropriateness of diagnostic decisions, although no sound supporting evidence exists (37). A histologic diagnosis of CMM in the prepuberal age should always be discussed with the pathologist and a second opinion by an expert pathologist should always be obtained (18). This study confirms the view that puberty is a crucial threshold (11) under which screening for CMM appears to be impracticable. At the same time, however, our results demonstrate that this view is largely disagreed upon.

High referral rates for dermatologic evaluation draw attention to persistent diagnostic challenges in primary care (21). Tailored educational programs are essential to strengthen general practitioners’ skills in reducing unnecessary referrals (38). Defensive medicine is certainly a supplementary driver of dermatologists’ decisions in the screening practice. No data specifically regarding CMM screening in primary care have so far been reported in Italy. However, institutional documents provide sufficient evidence for an unsustainably high frequency of lawsuits against medical institutions and doctors in many different specialties (39). The causes of defensive medicine have received great attention by health sociologists and law makers (39–41). The failures of medicine are presented by the media as the most visible aspects of medical practice and generate collective reactions (39). The current national regulations assign a central role to the guidelines set by the National Center for Clinical Excellence, Quality, and Security, adherence to which can lead to a reduction in medical malpractice claims (41).

Turning to research implications, future basic studies should cover the normal evolution of acquired naevi in children and adolescents (11, 12, 34) and the development of criteria to better identify which lesions are appropriate for skin biopsy versus clinical observation (23, 34). Probably, the most common reason for deciding for skin biopsy in children and adolescent is the presence of a ‘changing mole’ (11). In these patients, in fact, the clinical and dermoscopic detection of changes in the size and structure of naevi is poorly predictive of CMM (11, 12, 34, 35). Additional criteria for recommending biopsy and excision should be identified.

Finally, an effective risk communication, with a better understanding of the actual risk of CMM, may help patients and families to make more informed and appropriate decisions. In the last birth cohorts of Italians, the risk of CMM has started to decline (14), but this changing scenario is probably not perceived yet by the public.

### Strengths and weaknesses

This study has a high degree of novelty and a large population basis but suffers from limitations worthy of mention. The quality and completeness of data and the external validity of results are issues to consider. Firstly, we used an administrative healthcare database to identify routine diagnostic and procedural information. In many medical areas, original patient records are heterogeneously formatted and dispersed across multiple unconnected systems. Administrative healthcare data –a low-cost and widely available resource– are increasingly used for research purposes through the development of computation algorithms. These consist of sets of step-by-step instructions or procedures to extract information of medical interest. Validation studies have shown improvements in their accuracy in case ascertainment (42, 43). However, administrative healthcare data suffer from limitations in the specificity of disease coding and do not capture key information like, for example, the indication for a medical procedure (21, 44). Consequently, we were unable to distinguish office visits and biopsies done for melanocytic lesions from those done for other conditions affecting the skin.

Another problem is that we did not have access to data from the private sector, which conveys a risk of underestimating office visit rates and skin biopsy rates. It must be considered, however, that the prevalence of inpatient and outpatient treatment of CMM in the Emilia-Romagna Region is largely concentrated in public hospitals, as reported in another related article (22).

As regards the external validity of our observations, they cannot directly apply to populations served by different healthcare systems and with substantially different prevalence rates of benign and malignant pigmented skin lesions.

Some design issues also need to be addressed. First, we used biopsy –rather than excision– as main endpoint. In previous studies, either the former (12, 23) or the latter (7, 45) or both cumulated (9) were proposed. We believe that biopsy rate is a more sensitive measure of diagnostic scrutiny.

Second, we included *in situ* CMM in the definition of the target disease. From the perspective of this study, the detection of an *in situ* lesion appears to be a clinically valuable outcome, although it causes an overestimate of the yield of biologically significant disease.

And third, the study period was not recent. The research project of which this study is a part began in 2020. Breslow tumor thickness information –routinely not collected in Italy– was actively retrieved in 2021 for the years 2003-2017. This dataset was used for three reports prior to the present one (21, 22, 46) and not subsequently updated for budget constraints. In any case, the year 2020 and subsequent years could not be included in the analysis, because the COVID-19 pandemic led to delays and cancellations of nonessential medical care.

In conclusion, when screening children and adolescents for CMM, a trade-off between life-years saved and unnecessary biopsies is impossible to make because of the minimal prevalence and the ill-defined clinical/dermoscopic features of the disease. Further research is needed investigating the frequency, indications and results of dermatologic office visits and skin biopsies for CMM in young people both in Italy and Europe.

## Data Availability

The original contributions presented in the study are included in the article/**Supplementary Material**. Further inquiries can be directed to the corresponding author.

## Supplementary material

Supplementary Figure 1 Curves of average annual incidence rates of in situ **(A)**, invasive **(B)** and total **(C)** cutaneous malignant melanoma in the study period, per 100,000, by sex and five-year age group. Emilia-Romagna Region (northern Italy), 2003-2017.
