# Supplementary material for: Screening children and adolescents for cutaneous malignant melanoma: the impossible trade-off between life-years saved and unnecessary biopsies
Source: Front Oncol. 2026 Feb 23;16:1726375. doi: 10.3389/fonc.2026.1726375 (PMC12967977; doi:10.3389/fonc.2026.1726375)
Supplement: Supplementary Figure 1 — Curves of average annual incidence rates of in situ (A), invasive (B) and total (C) cutaneous malignant melanoma in the study period, per 100,000, by sex and five-year age group. Emilia-Romagna Region (northern Italy), 2003-2017. [file DataSheet1.pdf]

frontiers | Frontiers in Oncology

Screening children and adolescents for cutaneous malignant melanoma: the impossible trade-off between life-years saved and unnecessary biopsies

Lauro Bucchi[1], Silvia Mancini[1], Pietro Ceretti[2], Federica Zamagni[1], Emanuele Crocetti[1], Luigino Dal Maso[3], Stefano Ferretti[4], Flavia Baldacchini[1], Orietta Giuliani[1], Alessandra Ravaioli[1], Rosa Vattiato[1], Giuliano Carrozzi[5], Maria Michiara[6], Antonino Musolino[7,8], Fabio Falcini[1,9] and Ignazio Stanganelli[10,11,*]

[1] Emilia-Romagna Cancer Registry, Romagna Cancer Institute, IRCCS Istituto Romagnolo per lo Studio dei Tumori (IRST) Dino Amadori, Meldola, Forlì, Italy
[2] University of Parma, Parma, Italy
[3] Cancer Epidemiology Unit, Centro di Riferimento Oncologico di Aviano (CRO) IRCCS, Aviano, Italy
[4] Dipartimento di Medicina Translazionale e per la Romagna, University of Ferrara, Ferrara, Italy
[5] Emilia-Romagna Cancer Registry, Modena Unit, Public Health Department, Local Health Authority, Modena, Italy
[6] Emilia-Romagna Cancer Registry, Parma Unit, Medical Oncology Unit, University Hospital of Parma, Parma, Italy
[7] Breast & GYN Unit, Medical Oncology, IRCCS Istituto Romagnolo per lo Studio dei Tumori (IRST) Dino Amadori, Meldola, Forlì, Italy
[8] Department of Medical and Surgical Sciences, University of Bologna, Bologna, Italy.
[9] Cancer Prevention Unit, Local Health Authority, Forlì, Italy
[10] Skin Cancer Unit, IRCCS Istituto Romagnolo per lo Studio dei Tumori (IRST) Dino Amadori, Meldola, Forlì, Italy
[11] Dermatology Clinic, Department of Medicine and Surgery, University of Parma, Parma, Italy

*Corresponding author. Skin Cancer Unit, IRCCS Istituto Romagnolo per lo Studio dei Tumori (IRST) Dino Amadori, Meldola, Forlì, Italy.
E-mail: ignazio.stanganelli@unipr.it

Supplementary material

Supplementary FIGURE S1

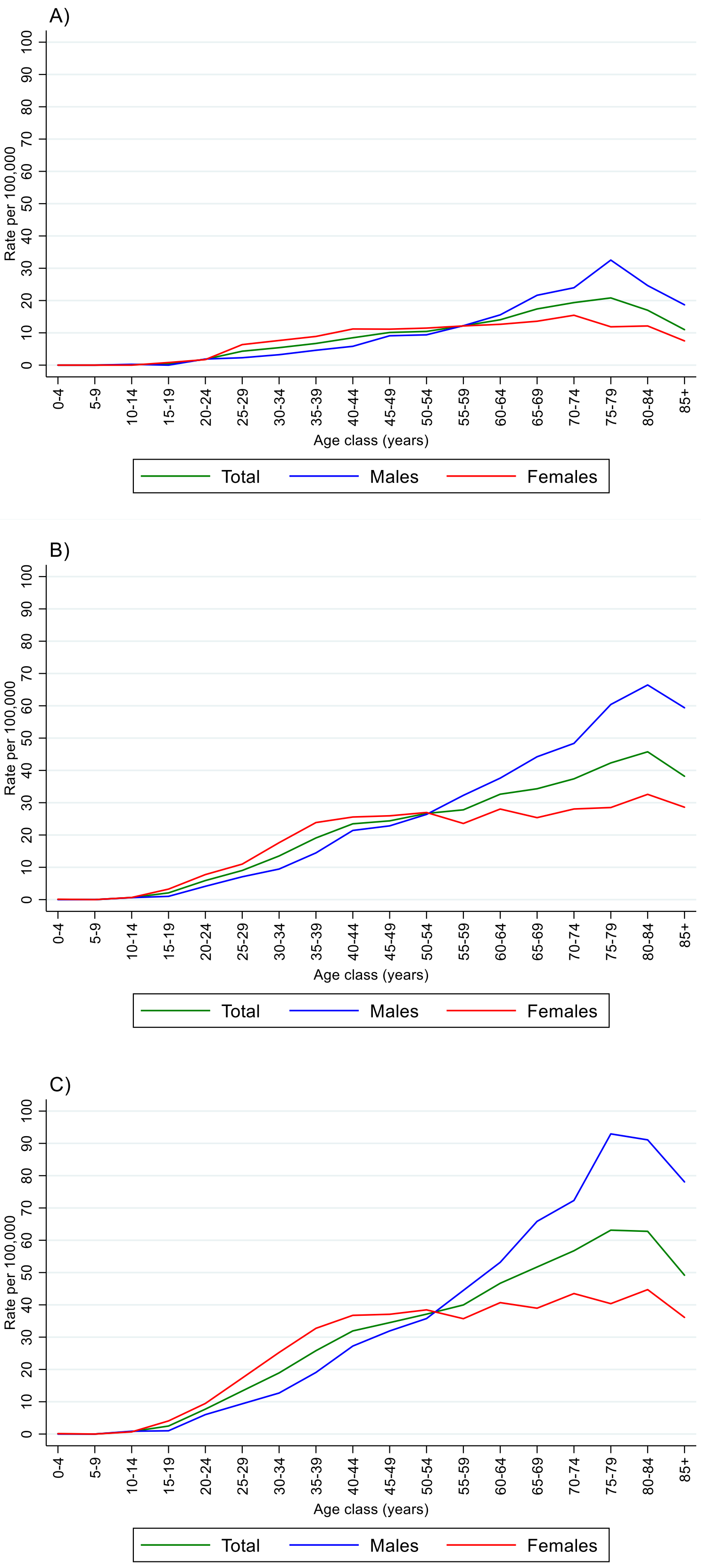

SUPPLEMENTARY FIGURE S1

Curves of average annual incidence rates of in situ (panel A), invasive (panel B) and total (panel C) cutaneous malignant melanoma in the study period, per 100,000, by sex and five-year age group. Emilia-Romagna Region (northern Italy), 2003-2017.
